# A digital spiritual health collaborative care model within accountable care organizations: a community case study

**DOI:** 10.3389/frhs.2026.1944486

**Published:** 2026-10-01

**Authors:** Alex Beth Schapiro, James E. Barr, Csaba Szilagyi

**Affiliations:** 1 Atlantic Health, Morristown, NJ, United States; 2 Atlantic Accountable Care Organizations, Morristown, NJ, United States; 3 College of Health Sciences, Rush University, Chicago, IL, United States

**Keywords:** Accountable Care Organizations (ACO), community health, digital spiritual care, population health, spiritual health, value-based care, collaborative care model, telechaplaincy

## Abstract

Spirituality is an established dimension of whole-person care and is increasingly recognized as a determinant of health, yet integrating spiritual care into value-based, population health care models, including Accountable Care Organizations (ACOs), remains rare. This descriptive community case study details the implementation of a novel digital spiritual health collaborative care model within ACOs in New Jersey, USA. The Atlantic Health ACO and Optimus Healthcare Partners ACO have 4,000 providers serving approximately 530,000 attributed patients in 11 different value-based payer arrangements, both risk and non-risk. Drawing on the interprofessional spiritual care framework, the program embedded a board-certified chaplain within the ACOs’ Integrated Care team, delivering specialist spiritual care to patients with chronic and serious illnesses via telephone and videoconference visits, with care documented directly in the shared electronic medical record. A two-item spiritual screening tool was administered by ambulatory care team members across primary care and specialty practices; 96/147 (65.3%) patients screened positive, and 58/96 (60.4%) enrolled in spiritual health services. Chaplain assessments identified grief and loss (62.1%), isolation (41.4%), existential concerns (31.0%), and conflicted or challenged belief systems (29.3%) as the most prevalent spiritual health concerns. The chaplain communicated spiritual concerns and spiritual health insights to care team members through consult notes. Nearly half of enrolled patients received referrals to supportive resources within the health system or the broader community through a developing Spiritually Integrated Network. Implementation was supported by targeted staff education on spiritual health and interprofessional spiritual care. The program demonstrated operational feasibility and meaningful patient engagement through a Patient-Centered Medical Home (PCMH) primary care infrastructure. The PCMH principles of person-centered orientation, team-based care, care coordination, and quality-focused value-based incentives offered a practical and supportive framework for embedding spiritual health into ambulatory workflows. These findings suggest that ACOs operating within PCMH models may be particularly suited for integrating spiritual care. This work addresses a critical gap in population health practice and points toward the potential contribution of spiritual health programs to the Quintuple Aim, warranting further research.

## 1 Introduction

Spirituality is an essential resource for coping with illness and is increasingly recognized as a significant determinant of health, underscoring the need for greater integration of spiritual care into routine healthcare and public health (1, 2). Additionally, healthcare delivery in population health and value-based models, such as Accountable Care Organizations (ACOs), has expanded in the United States, focusing on improving the health of designated populations and reducing costs through coordinated, high-quality, and person-centered care (3, 4). However, ACOs have rarely integrated spiritual care, and documented examples in ACO and population health settings remain limited (2, 5).

In ACOs, healthcare providers group together to deliver coordinated services to a specific population within a geographic area or based on a particular condition, with care management fees and financial incentives directly linked to health outcomes, quality metrics, patient experience, and cost reduction, thereby generating value rather than volume of healthcare services (4). ACOs have sought to achieve the Triple Aim: “improving the experience of care, improving the health of populations, and reducing per capita costs of health care” (6), which has since expanded to include a fourth and fifth aim: enhancing healthcare workers’ well-being (7) and advancing health equity (8). Research shows that ACOs are effective in reaching their goals, particularly with respect to the triple aim (9–11). Their success is closely tied to the use of digital and data-driven care delivery through collaborative, integrated care teams.

The person-centered, integrated, and collaborative care environment of ACOs can be seen as suitable for integrating spiritual care to attend to the whole person, including the spiritual dimension, as has been the case in other clinical settings, such as palliative care (12, 13). The interdisciplinary consensus defines spirituality in healthcare as an essential part of being human, including seeking purpose and meaning and transcendence; experiencing connection to self, others, and something greater than the self or the sacred; and being expressed through religious or non-religious beliefs, values, or practices (12).

Multidisciplinary consensus and research indicate that spiritual care contributes to compassionate, whole-person care, quality of life, patient outcomes and experience, and healthcare provider well-being (1, 12, 14–17). Specifically, in ambulatory contexts (e.g., primary care, outpatient palliative care, and outpatient oncology), the delivery of chaplain and spiritual care interventions is feasible and acceptable, and is linked to positive spiritual and psychosocial outcomes (15).

Healthcare chaplaincy clinical practice and workflows have largely not evolved from volume-based approaches traditionally used in inpatient hospital settings to value-based and population health care models. This is even as patient care grows exponentially in ambulatory care settings, potentially resulting in a shrinking role for spiritual care in daily healthcare practice if the field is unable to adapt. Thus, innovative projects, such as the one described in this paper, and subsequent research are needed to explore the potential impact of integrating spiritual care into ACOs and value-based care settings and its contributions to the Quintuple Aim.

Patient care and teamwork in ACOs increasingly rely on digital health infrastructure, including virtual patient care delivery, electronic health records, data-sharing systems, and population health analytics platforms, to support coordinated, value-based care (18). However, little attention has been paid to integrating spiritual care specialists and models that promote spiritual health and address spiritual needs in digital health settings (19), despite patients finding digital spiritual care acceptable and important (20, 21). A population survey found that 26.3% of respondents rated the availability of spiritual care through digital means as important or very important in healthcare settings (21). Adopting digital modalities is essential to bringing spiritual health programs into these care environments. Digital spiritual care also enables the incorporation of processes and chaplains across all areas of care, communication, and data analysis for ACO healthcare teams, presenting unprecedented opportunities for collaboration and integration. Thus, our program was intentionally designed to leverage technologies for patient care, collaboration, and care management.

This project aimed to demonstrate the value and feasibility of a digital spiritual health collaborative care program within the Atlantic ACOs to address spiritual health and contribute to whole-person care for a growing patient population with chronic and serious illnesses. The purpose of this community case study is to describe the program, our experiences with its implementation and care delivery, and the insights and implications that arose.

## 2 Context

The digital spiritual health collaborative care model was implemented within Accountable Care Organizations (ACOs) affiliated with Atlantic Health in Northern New Jersey, United States, from September 2023 to October 2024. The Atlantic Health ACO and Optimus Healthcare Partners ACO have 4,000 providers serving approximately 530,000 attributed patients in 11 different value-based payer arrangements, both risk and non-risk. The ACOs operate within the Patient-Centered Medical Home (PCMH) framework, an evidence-based primary care model designed to improve quality, efficiency, and patient experience by delivering care that is personalized, comprehensive, coordinated, accessible, and focused on quality and safety (22, 23). Although this model is designated as patient-centered, this paper consistently uses the term person-centered because it reflects developments in healthcare delivery that conceptualize the individual holistically and in context, beyond their clinical status as a patient. This maintains alignment with the PCMH tenets that center the whole person (22, 23).

Atlantic Health's prior success in implementing collaborative care approaches, including integrated behavioral health embedded within primary care workflows, supported our spiritual health implementation by establishing structured screening-to-referral pathways, interdisciplinary documentation within the electronic medical record (EMR), and coordinated communication across care teams. These structural elements, associated with PCMH transformation and improved care integration, emphasize whole-person orientation, team-based care, longitudinal responsibility for patients across settings, and systematic use of health information technology (22).

The spiritual health program was intentionally aligned with the defining PCMH criteria. It was person-centered (termed patient-centered in the original PCMH criteria as noted above), addressing patients’ values, coping frameworks, and spiritual and existential concerns as integral to health, and helping them access supports in the health system and the community. It was comprehensive, integrating spiritual assessment with physical and behavioral domains. It was coordinated, with Spiritual Health Insights documented in the shared EMR and communicated to interdisciplinary clinicians. It was accessible, leveraging digital health delivery and transitions-of-care referral pathways to reduce barriers to engagement. Finally, it was focused on quality and safety, incorporating structured spiritual distress screening and reinforcing chronic disease follow-up and advance care planning within longitudinal workflows. Consistent with PCMH principles, the model operated within a team-based, technology-enabled infrastructure to support whole-person, value-based care (22, 24).

The spiritual health program was implemented as a demonstration project funded through the ‘Advancing Spiritual Care in Everyday Clinical Practice’ initiative of The George Washington University Institute for Spirituality & Health (GWish), supported by a grant from the John Templeton Foundation. This initiative supported innovative projects to demonstrate the feasibility and value of interprofessional spiritual care clinical models across various healthcare settings, ultimately contributing to pathways for sharing best practices and for more widespread, standardized integration of interprofessional spiritual care. It was carried out as a program implementation and demonstration project across the Atlantic ACOs. This work was conducted as a program evaluation, and IRB review was not required. Program evaluation drew on de-identified EMR data and chaplain documentation across three domains: patient screening and enrollment, spiritual health concerns, and referrals to supportive resources, using a quantitative, descriptive design. Data were tabulated in Microsoft Excel, and descriptive statistics (frequencies and percentages) were calculated using the same software.

### 2.1 Interprofessional spiritual care model

Another important context is that this program drew on the interprofessional spiritual care model in its design. This collaborative spiritual care approach brings together spiritual care specialists (i.e., chaplains) and spiritual care generalists (i.e., non-chaplain care team members) to share responsibilities and work closely to attend to patients’ spirituality and address their spiritual health (25, 26). Within their respective scopes of practice, healthcare professionals use basic, generalist skills to recognize patients’ spiritual needs, screen for spiritual distress, and, when appropriate, take a spiritual history. When spiritual needs or distress are identified, team members refer the individual to the specialist chaplain for a more comprehensive spiritual assessment and care. Ongoing communication and consultation between the chaplain and healthcare providers ensure that the patient's needs, preferences, and goals, along with the chaplain's recommendations, are incorporated into the care plan.

To support the practical application of this model, the program also included ACO-wide provider education, including webinars that introduced and reinforced the generalist–specialist framework, clarified roles and referral pathways, and built clinician confidence in addressing spiritual needs within routine care. Finally, both the project leadership (co-led by a family medicine physician and the Chief Medical Officer [J.E.B.], and by the program manager and clinical chaplain [A.B.S.]) and the program's design and implementation mirrored this interprofessional, collaborative approach.

## 3 Programmatic elements of the digital spiritual health collaborative care model

The program embedded the chaplain into the Atlantic ACOs’ Integrated Care team to provide specialist spiritual care to patients and their family caregivers. While chaplains served at Atlantic Health hospitals, it was the first effort to provide spiritual care as part of the system's robust ambulatory care services. The novelty of this program lay in its integration into the ACOs’ value-based, population-health focused healthcare delivery. ACO patients receiving care from the Atlantic ACOs’ primary care physicians were eligible to receive services. For this initial phase, one board-certified chaplain (A.B.S.) provided specialist spiritual care, consistent with established qualifications and certification standards for healthcare chaplains in the United States (27), with the aim of developing an ACO chaplain team in the future.

The program initially received referrals for attributed ACO patients from the Atlantic Health Overlook Medical Center (OMC) across multiple ambulatory settings. Other referral sources were added, including OMC's Cancer Center, outpatient Palliative Care, and Care Coordination services. The Care Coordination pathway included social workers, Transitions of Care (TOC) nurses supporting high-risk patients during the first 30 days post-discharge, and ambulatory care coordinators and nurses providing continued longitudinal follow-up beyond the initial transition period. This multi-entry referral structure enabled the spiritual health program to engage patients during periods of acute post-discharge vulnerability and throughout sustained chronic disease management. Figure 1 presents a flow diagram of the spiritual health program. Supplementary Table 1 provides a composite case example of a patient journey across the elements of the spiritual health program.

**Figure 1:**
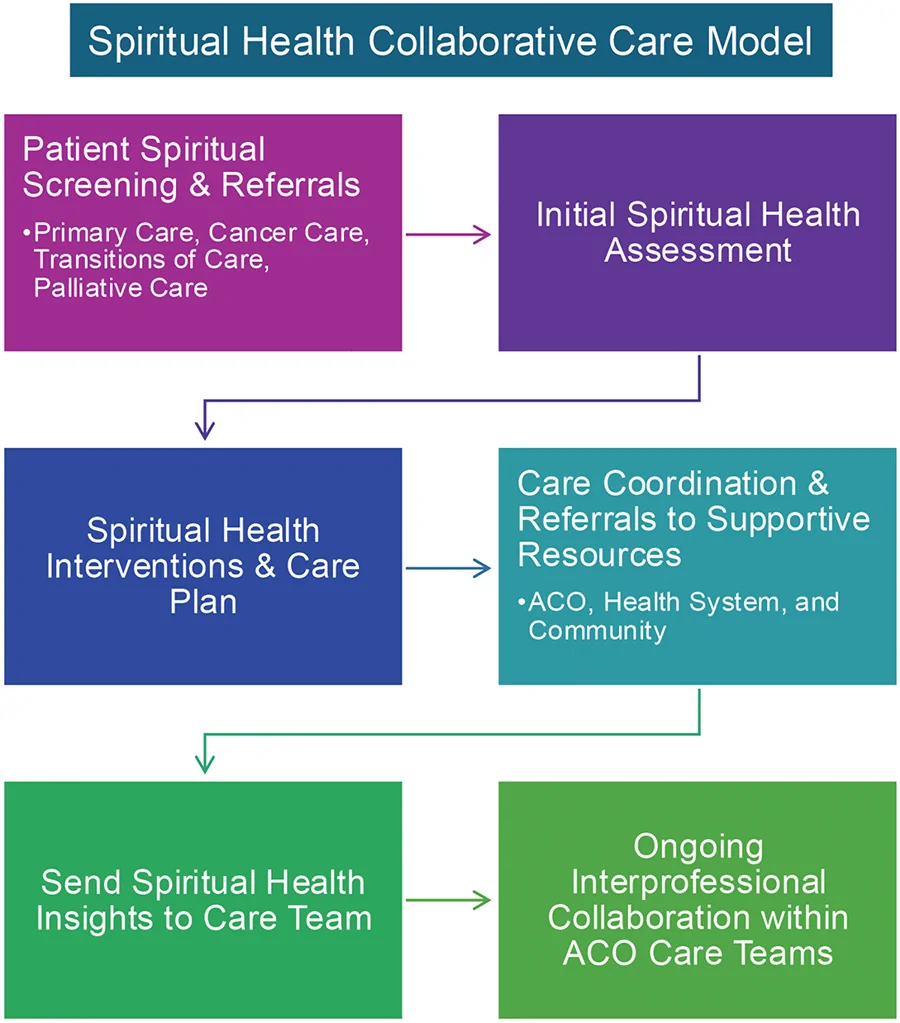
Program workflow diagram of the digital spiritual health collaborative care model. ACO, Accountable Care Organization.

### 3.1 Spiritual screening

The program developed and used a two-item spiritual screening (called SHQ-2) to identify patients who may desire or benefit from spiritual care services. The tool was developed by Dr. James Barr and Rev. Stephen Faller, a spiritual care educator at Atlantic, to be religiously and spiritually inclusive, accessible across belief systems, and oriented toward patient self-interest rather than crisis-based identification of distress. The SHQ-2 included two questions: “Is philosophy, religion, or spirituality important to you?” and “Would you be interested in resources that can help you with your spiritual needs?” In determining the appropriate screening tool, we recognized the lack of tools specifically designed for population health and ambulatory care. The development and validation of evidence-based screening tools that align with longitudinal patient experiences and screening workflows in these contexts remain needed.

The brief screening allowed any care team member to efficiently identify potential spiritual concerns and the need for a chaplain referral, and it could be embedded in existing screening and clinical workflows in the EMR. We located the SHQ2 screen next to the already established PHQ2 for depression screening. Ambulatory care teams (physicians, nurses, physician assistants, social workers, and care coordinators) administered this screening to ACO patients. Care team members then sent referrals for patients who screened positive (answered yes to both questions) via the EMR and other channels to the ACO chaplain for an initial spiritual assessment and care plan development (also see Supplementary Table 1).

Of 147 patients, 96/147 (65.3%) were screened positive by care team members. Then, 58/96 (60.4%) patients enrolled in the spiritual health program, involving spiritual assessment, care planning, and chaplain visits. Table 1 presents these 58 patients’ demographic characteristics.

**Table 1:** Demographic characteristics (*N* = 58).

Characteristic	*N* (%)
Age, median (IQR) range	68 (18) 36–94
Gender^a^	
Female	36 (62.1)
Male	22 (37.9)
Race^a^	
White, non-Hispanic or Latino	38 (65.5)
Black or African American, non-Hispanic or Latino	14 (24.1)
White, Hispanic or Latino	2 (3.4)
Other, Hispanic or Latino	2 (3.4)
Other, non-Hispanic or Latino	1 (1.7)
Unknown	1 (1.7)
Religious affiliation^a^	
Christian affiliations	45 (77.6)
Catholic	23 (39.7)
Christian	8 (13.8)
Baptist	3 (5.2)
Episcopalian	2 (3.4)
Methodist	2 (3.4)
Assembly of God	1 (1.7)
Greek Orthodox	1 (1.7)
Lutheran	1 (1.7)
Pentecostal	1 (1.7)
Presbyterian	1 (1.7)
Protestant	1 (1.7)
Seventh-day Adventist	1 (1.7)
Jewish	7 (12.1)
Muslim	2 (3.4)
None	1 (1.7)
Unknown	3 (5.2)

Percentages are based on *N* = 58 patients enrolled in the spiritual health program.

a Demographic analyses were constrained by the predefined categories available in the electronic medical record (EMR). Gender, race, ethnicity, and religion/spirituality were drawn directly from EMR data fields. Gender was recorded in a single binary field (male/female); these terms reflect biological sex classifications rather than patients’ gender identities. Race, ethnicity, and religion/spirituality were likewise limited to the EMR's administrative categories, which do not fully capture patients’ cultural or spiritual self-understandings.

### 3.2 Spiritual assessment, interventions, and care planning

While the brief spiritual screening serves a triage function, spiritual assessments are more dynamic and in-depth, address spiritual distress, needs, and resources, and guide interventions and care plans (28). The chaplain connected with patients by telephone or videoconference to conduct spiritual assessments and develop spiritual care plans. These plans included evidence-based spiritual interventions guided by the chaplain, as well as referrals within the health system and to the community (see Supplementary Table 1). The program provided patients with an initial spiritual health assessment, followed by additional visits based on patient preferences and needs.

The patient and the chaplain collaboratively determined the appropriate visit cadence, enabling a personalized approach to spiritual care. The frequency and duration of spiritual health visits varied based on patient preference, availability, and the acuity of spiritual health needs, and were aligned with the patient's healthcare journey. Some patients preferred weekly or biweekly sessions with more focused, in-depth spiritual counseling lasting 45 to 50 minutes, whereas others preferred monthly check-ins with shorter 20- to 30-minute sessions.

The program's visit frequency was not constrained by insurance-based limits, which allowed patients to determine the pace and duration of spiritual health engagement that best suited their needs. Service utilization showed that 19 of 58 patients (32.8%) had one spiritual health visit, 16 (27.6%) received two to three visits, and 23 (39.7%) sought four or more visits with the chaplain. Of the 19 patients with a single visit, 12 (63.2%) were planned for a brief, single-visit engagement based on the spiritual assessment and did not require further follow-up, having received sufficient support or been connected to further resources, while 7 (36.8%) did not continue with the established care plan. Additionally, caregiver support for family caregivers was included in eight patient cases (13.8%).

The chaplain most frequently identified spiritual health concerns among enrolled patients (*N* = 58) as grief and loss (62.1%, *n* = 36), isolation (41.4%, *n* = 24), existential concerns (31.0%, *n* = 18), conflicted or challenged belief systems (29.3%, *n* = 17), despair or hopelessness (27.6%, *n* = 16), and reconciliation (25.9%, *n* = 15) (Table 2), using categories outlined by a consensus conference led by Puchalski and colleagues (29). Although these categories were not intended to capture the complexities of one's spiritual health, using clear language to identify core spiritual concerns and describe the nature of the distress enhanced communication and collaboration with the interprofessional team. The identified concerns guided spiritual health interventions, care plans, and referrals. This approach also helped connect patient concerns to broader dynamics in health and chronic disease management. Additionally, 24.1% of spiritual health visits included instruction and support for meditation or contemplative practices.

**Table 2:** Spiritual health concerns assessed by the chaplain (*N* = 58).

Spiritual health concerns^a^	Patients with the concern, *N* (%)^b^	Patients for whom it was the primary concern, *N* (%)
Grief/loss	36 (62.1)	12 (20.7)
Isolation	24 (41.4)	6 (10.3)
Existential concerns	18 (31.0)	2 (3.4)
Conflicted or challenged belief systems	17 (29.3)	8 (13.8)
Despair/hopelessness	16 (27.6)	5 (8.6)
Reconciliation	15 (25.9)	9 (15.5)
Guilt/shame	12 (20.7)	3 (5.2)
Anger at God or others	9 (15.5)	2 (3.4)
Concerns about relationship with the transcendent or sacred	8 (13.8)	3 (5.2)
Religious/spiritual struggle	8 (13.8)	3 (5.2)
Abandonment by God or others	7 (12.1)	4 (6.9)
Religious-specific practices	4 (6.9)	1 (1.7)

Percentages are based on *N* = 58 patients enrolled in the spiritual health program.

a Categories of spiritual health concerns were adapted from Puchalski et al. (29).

b The chaplain could select more than one option, so the percentages may sum to more than 100%.

### 3.3 Healthcare team integration and collaboration

With patient consent, the chaplain communicated patients’ personalized spiritual health care plans to their clinical teams to enhance integration and collaboration within the care team. The novel chaplain workflow included patient encounter documentation designed as a consult note for the referring clinical care teams. The top of the note contained the most pertinent information for the care team: (1) primary and secondary spiritual concerns (29) (Table 2) and (2) three brief “Spiritual Health Insights” that could be readily integrated into patient care delivery by the referring clinical team (Supplementary Table 1). The Spiritual Health Insights reflected patients’ spiritual strengths and challenges that impact the patient's experience of healthcare.

To support program implementation and the adoption of spiritual screening, J.E.B and A.B.S. provided targeted education on spiritual health and interprofessional spiritual care. Team members across all ambulatory care locations received training to administer the SHQ-2. Training sessions incorporated materials from the ISPEC^©^™ curriculum, which centers on the generalist-specialist model of spiritual care (30). Emphasis was placed on three key areas: foundational research on the benefits of spiritual care in healthcare, identifying eligible patients and signs of spiritual distress, and cultivating clinicians’ self-awareness and comfort with spirituality as an important baseline.

### 3.4 Referral to supportive and community resources

Another area of innovation was an expanded integrated care approach that includes referrals to the Spiritually Integrated Network (SpIN), social services, behavioral health, the caregiver center, humanistic medicine, and relevant support groups, as well as reconnecting patients with their care teams. The SpIN, inspired by the Clinically Integrated Network (CIN) model, is a developing network of spiritual care resources within the larger community. The SpIN was created through a collaboration between ACO Spiritual Health and the Community Health department at Atlantic Health. Nearly half of the patients receiving spiritual health services (48%, *n* = 28) had spiritual care plans with one or more referrals to supportive resources within Atlantic Health and into the larger community. It led to 54 distinct referrals, out of which 44.4% were to the SpIN (27.8% to faith communities), 14.8% to social work, and 13.0% to advance care planning resources (see additional referrals in Table 3).

**Table 3:** Referrals to supportive and community resources (*N* = 54).

Supportive and community resources	Referrals, *N* (%)
Spiritually Integrated Network (SpIN)	24 (44.4)
Faith community support	15 (27.8)
Illness-specific support	4 (7.4)
Caregiver support in community	3 (5.6)
Grief & bereavement support	2 (3.7)
Social work	8 (14.8)
Advance care planning resources	7 (13.0)
Caregiver center at Atlantic Health Overlook Medical Center (OMC)	3 (5.6)
Humanistic medicine at OMC	3 (5.6)
Inpatient chaplaincy at OMC	3 (5.6)
Reconnecting patients with care team	3 (5.6)
Behavioral health	2 (3.7)
New referrals to palliative care	1 (1.9)

Percentages are based on 54 distinct referrals made across all patients with a spiritual care plan. Patients could receive more than one referral. Faith community support, illness-specific support, caregiver support in community, and grief and bereavement support are referral subcategories within the Spiritually Integrated Network (SpIN).

## 4 Discussion of practical implications and lessons learned for future applications

This community case study describes the successful implementation of a novel digital spiritual health collaborative care model within an ACO setting. We observed that the program is feasible, demonstrating a strong practical and operational fit and effective technical and patient-care integration across Atlantic Health ACOs. Patient engagement also suggested acceptability and demand for spiritual health services. Aligning the spiritual health program with defining PCMH tenets (person-centered, comprehensive, coordinated, accessible, and quality-focused) (22, 23) provided structural support and an operational template for embedding it in ambulatory care workflows. This suggests that ACOs operating within PCMH models may be particularly well suited for integrating spiritual health (24), given their existing infrastructure for team-based, technology-enabled, whole-person care. More broadly, this work aligns with the recent call for “a reimagined clinical and public health system” in which “spiritual factors would be routinely considered in creating person- and community-centered policy and practice” (2). It addresses recommendations to recognize spirituality as a key social factor influencing health outcomes, to educate healthcare professionals about spirituality and health, and to provide spiritual care by chaplains within healthcare systems (1, 2).

### 4.1 Patient engagement, preferences, and needs

Strong service uptake and patient engagement, with nearly two-thirds of patients seeking more than one visit, indicate that a considerable portion of patients found spiritual health services acceptable and useful. Within the single-visit subgroup, most patients had concluded care by design after receiving sufficient support in a single encounter, rather than discontinuing an active care plan. This distinction suggests that the single-visit figure reflects patient-directed care as much as it does attrition. Patients exhibited a wide range of spiritual health concerns, with grief and loss interwoven as a prominent experience in dealing with chronic and serious illnesses. Our findings are consistent with a study of 1,249 outpatient oncology patients, which found that 30% reported at least one spiritual concern, most frequently related to family relationships (75%) and fear of death (28%) (31). Researchers also noted high feasibility and acceptability, with chaplains contacting 61% of those who screened positive and providing interventions to 48% (20). Similarly, a health system that incorporated spiritual care into its remote patient monitoring program reported that 35% of 1,453 patients and 9% of 2,354 patients screened positive for spiritual distress across two geographic regions (32). Our program furthered understanding of the prevalence and nature of patients’ spiritual needs in ambulatory care settings.

#### 4.1.1 Implications for spiritual screening and assessment

Further work is needed to develop spiritual screening and assessment tools, data-collection instruments, and digital work platforms tailored for integrating spiritual care into ambulatory care and population health contexts. Healthcare professionals can use one- or two-item tools to quickly screen for spiritual concerns and determine whether a referral to a chaplain is warranted. Methods and tools for spiritual screening vary widely, from validated instruments (33) to those created by local programs (such as the SHQ-2). Approaches also differ in their goals: whether to detect spiritual distress or to identify spiritual needs and concerns among patients. This, along with our experience with the program, underscores the need to develop and evaluate spiritual screening tools that are valid, demonstrate adequate sensitivity and specificity, and are appropriate for ambulatory care settings.

Spiritual health programs should also develop strategies and best practices for incorporating spiritual screening into clinical and EMR workflows and healthcare systems (34). Digital tools for chaplains should align with the workflows of related healthcare professions, such as ACO-based case management, which focus on value-based outcomes. Chaplain workflows could also include comprehensive social determinants of health (SDOH) screening tools for more robust collaboration with care coordination and community health.

Chaplains conduct spiritual assessments that are more dynamic and in-depth, address spiritual distress, needs, and resources, and guide interventions and care plans (28). Existing spiritual assessment tools range from generic to clinical-specialty models (35–38), yet none are designed for population health settings. Our program underscores the critical need to develop dedicated spiritual assessment tools for ambulatory patients with chronic illnesses.

### 4.2 Digital health and EMR integration

While individual exemplars of digital spiritual health delivery exist in the United States (39), this modality remains in early stages within the field of spiritual care and chaplaincy. Our experience mirrored that digital spiritual health programs often need to address factors at the individual level (e.g., clinical practice changes, workflow, interventions, and the provider-patient connection), the organizational level (e.g., strategic alignment and adoption), and the population level (e.g., shifts toward ambulatory and chronic illness care, socioeconomic and cultural barriers, and patient acceptance and utilization) (40). In our case, the use of digital technologies was not only essential but also accelerated implementation and enhanced the adoption and effectiveness of the spiritual health program. As healthcare delivery and teamwork have become primarily digital at Atlantic Health ACOs, designing a digital-first spiritual health program has supported communication, engagement, and collaboration with patients and teams, as well as organizational alignment.

Barriers to implementation included variability and gaps in EMR designs. Specifically, the EMR workflow for enabling SHQ-2 in ACO patient records and for sending referrals and reports to the chaplain for contact and follow-up proved more complicated and time-consuming than expected. Additionally, there were a few referral triggers based on real-time health data relevant to patients’ spiritual health, aside from recent hospitalizations of ACO patients. Useful triggers in patients’ health data might have included new diagnoses or prognoses, missed appointments, non-compliance with care plans and treatments, or referrals to palliative care, prompting consideration of whether spiritual screening, spiritual assessment, or chaplain contact was necessary.

The most surprising use of digital technology in our project was text messaging. Chaplaincy has primarily relied on telephone- or videoconferencing-based communication and interventions for patient care (39), often overlooking the full spectrum of digital health technologies, including messaging, online chat, asynchronous interactions, and eHealth and mHealth applications. In our program, patient-chaplain communication was facilitated via secure text messaging. Very few patients preferred email or phone calls to stay in touch between scheduled visits or to send relevant spiritual resources. Many patients favored text messaging to manage future appointments and, increasingly, to seek spiritual support outside virtual patient care times. This included communication about recent changes in prognosis, pre- and post-medical-procedure check-ins, and sharing personal spiritual insights as they emerged between scheduled visits. Finally, we observed that these communication modalities were not only practical and necessary but also offered unexpected therapeutic benefits to spiritual care recipients.

### 4.3 Spiritual care clinical practice implications

In its most common form, spiritual care clinical practice has evolved to support hospitalized patients facing acute, often critical illnesses within a limited timeframe. In our program, however, longer-term supportive relationships span months, encompassing the ups and downs of life and health experiences, as well as changes in relationships and resources. The frequency, nature, and intensity of spiritual care encounters may vary throughout a person's health journey. Nevertheless, the ongoing patient-provider relationship persists and continues to develop. This creates new opportunities and challenges for chaplaincy practice within the ACO context.

Chaplains should look to resonant professions, such as psychology and social work, for guidance on navigating the complexities of boundaries, self-disclosure, and termination in this new ambulatory context. This can help chaplains establish best practices to maintain the integrity and effectiveness of spiritual care across longer-term relationships with patients. Further work is needed to understand the nuances of ambulatory spiritual care and to determine the optimal structure for chaplain visits, including therapeutic approaches, interventions, duration, and frequency. Additionally, the distinct spiritual care practice should inform chaplaincy training, ensuring that chaplains are properly equipped to build and sustain therapeutic spaces for long-term spiritual care relationships with patients in this setting. Stakeholders should define skills specific to delivering and integrating spiritual care in population health and community health environments (41) and create the necessary training programs (e.g., specialized fellowship opportunities) beyond basic clinical chaplaincy education.

### 4.4 Health system integration

A primary facilitator for launching a spiritual health program within the Atlantic Health ACOs has been the full support of ACO leadership. The program has benefited from their clinical insights, administrative guidance, and troubleshooting related to the integration into this distinct ambulatory care model. ACOs, including Atlantic ACOs, have served as sites of significant healthcare innovation and offer supportive infrastructure for early innovation and implementation, including in-house data analysis. Barriers have included challenges with integrating spiritual health workflows into the ambulatory EMRs, EMR system variability across affiliated primary care practices, inconsistent clinical care team buy-in, developing and testing a sustainable financial model using a discrete, trackable data model, and limited access to technologies to effectively coordinate interdisciplinary care across settings.

### 4.5 Future directions and opportunities

Stronger integration of data across spiritual health, SDOH, health outcomes, and key value-based care metrics is crucial. This could yield insights into viable financial models for spiritual health in the current healthcare climate, where clinicians are shifting away from fee-for-service models. The potential reduction in overall care costs, tracked through the per-member-per-month (PMPM) metric, a key indicator in value-based ACOs, could serve as a compelling measure of effectiveness and success. Incorporating comprehensive SDOH assessments and care plans may help integrate spiritual health more effectively into existing community health efforts. For example, leveraging platforms with closed-loop SDOH referral capabilities, such as Unite Us (42), could organize, make accessible, and track spiritual support resources for patients and family caregivers.

Emerging opportunities include using AI-enabled and technology-supported workflows to scale screening, triage, and intervention. These approaches could enable continuous, low-burden identification of spiritual and psychosocial needs, allowing patients to be proactively flagged for spiritual care and advance care planning alongside longitudinal chronic care management. By shifting screening and monitoring upstream, these models could reduce clinician burden while improving the timeliness, accuracy, and appropriateness of referrals.

System-wide ACO initiatives, such as improving advance care planning workflows and medication adherence, offer important opportunities for integration. For example, increased clinician–chaplain collaboration in advance care planning could enhance annual wellness visits, strengthen attention to psychosocial, spiritual, and health equity factors, and reduce the burden on physician practices. Spiritual health programs could collaborate closely with clinical pharmacists and care teams to manage polypharmacy by addressing the beliefs, motivations, and barriers that influence adherence and decision-making. Embedding spiritually informed approaches within these efforts may strengthen patient engagement, support goal-concordant care, and enhance the effectiveness of existing population health strategies. The potential integration and impact of spiritual health services in these areas warrant further investigation.

Further research and program evaluation, incorporating perspectives from patients, family caregivers, and clinicians, should collect mixed-methods data on experiences, needs, preferences, and outcomes in spiritual health programs. A natural next step is to analyze how spiritual health programs can contribute to the Quintuple Aim (8), including improvements in patient experience, population health, healthcare costs, healthcare workers’ well-being, and health equity. Research should explore how spiritually integrated, person-centered care, delivered through clinician-chaplain collaboration, could improve patient experience, outcomes, and clinician well-being and satisfaction.

### 4.6 Limitations and methodological constraints

This community case study reflects our team's experiences and may not be directly applicable to other settings or types of spiritual health initiatives. While our descriptive approach aligns with the community case study method, we did not use a control group or formal feasibility or satisfaction instruments, nor did we conduct pre- and post-analysis to demonstrate program outcomes. We recognize that additional longitudinal data from a larger patient sample are needed to better evaluate the acceptability and impact of the digital spiritual health collaborative care model. Percentages reported throughout this manuscript reflect the modest sample size (*N* = 58), meaning that a single patient's shift can change the reported percentages by roughly 1.5 to 2 percentage points. Additionally, data analysis has been limited by EMR data that do not fully capture demographic details such as religious/spiritual, gender, ethnic, racial, and cultural identities. Future research designs should prioritize additional data-collection methods and linking to relevant existing datasets within ACOs to gain more comprehensive and accurate insights into the acceptability and impact of interventions across diverse populations.

### 4.7 Conclusion

This project demonstrates that integrating spiritual health services into value-based ACOs is operationally feasible, and patient engagement patterns suggest the program was meaningful to those it served. The supportive infrastructure may already exist in many ACOs operating within person-centered PCMH models. Barriers, such as EMR workflow gaps, the absence of validated ambulatory screening tools, and inconsistent clinician buy-in, are present but addressable. This work ultimately shows that spiritual health can be embedded within population health delivery in a way that is coordinated, digitally enabled, and responsive to the concerns patients bring to their care. It also, in practical terms, addresses calls to treat spirituality as a key determinant of health that warrants routine consideration in person- and community-centered care, professional education, and chaplain integration across health systems (1, 2). Realizing that potential at scale will require rigorous evaluation, purpose-built tools, and a spiritual health field willing to evolve beyond inpatient healthcare settings.

## Data Availability

The datasets presented in this article are not readily available because the datasets contain protected health information from patients’ electronic medical records, and institutional approval and further de-identification would be required prior to any external sharing. Requests to access the datasets should be directed to AS, alex.schapiro@atlantichealth.org.
