# Supplementary material for: A digital spiritual health collaborative care model within accountable care organizations: a community case study
Source: Front Health Serv. 2026 Oct 1;6:1944486. doi: 10.3389/frhs.2026.1944486 (PMC13668842; doi:10.3389/frhs.2026.1944486)
Supplement: Supplementary file 1 [file Datasheet1.pdf]

Supplementary Material

Supplementary Table 1. Composite spiritual health patient case example

Spiritual Health Program Elements	Composite Patient Case Example*
Spiritual Screening and Referral	Mrs. A was referred to the Spiritual Health program by the Transitions of Care (TOC) nursing team following hospitalization for uncontrolled hypertension. During routine post-discharge outreach, the TOC nurse administered the SHQ-2 spiritual distress screening questions. Mrs. A screened positive, prompting an electronic referral through the EMR. No advance care planning documentation was present in the electronic health record.
Spiritual Assessment and Care Plan	Within one week of discharge, the chaplain conducted a 30-minute video visit and completed a comprehensive spiritual assessment.<br>Mrs. A, a full-time working parent of two young children, reported significant caregiver strain following her mother's cancer diagnosis. Her mother had moved into Mrs. A's home due to escalating care needs. Mrs. A described a longstanding involvement in her Pentecostal church community, including prior leadership in educational programming. Since her mother's diagnosis, she struggled to recalibrate sustainable boundaries between caregiving, employment, parenting, and church commitments.<br>Care plan included meeting for an initial set of four weekly spiritual counseling visits with the chaplain, with goals to explore boundaries within family and church roles, grief processing (initially anticipatory), and faith-integrated contemplative practice for spiritual nourishment and resilience in her healthcare journey.
Spiritual Concerns and Spiritual Health Insights	The chaplain documented a primary spiritual concern of conflicted or challenged belief system and secondary concern of grief and loss and isolation in the EMR consult note.<br>Spiritual Health Insights documented for the care team:<br>1. Mrs. A is experiencing significant role strain in caring for her mother, parenting, employment, and church commitments, contributing to emotional and spiritual distress.<br>2. Anticipatory grief related to her mother's cancer diagnosis is present, with limited time and space to process loss and meaning outside of caregiving responsibilities.<br>3. Strong religious identity is a key internal resource; the patient may benefit from support in integrating spiritual practices and theological reflection into her healthcare experience to sustain resilience and engagement.
Ongoing Spiritual Care and Interventions	Mrs. A initially engaged in four consecutive weekly video visits to address acute caregiver stress and anticipatory grief. As stability improved, visit frequency transitioned to monthly follow-ups. After her mother's death,

	visits increased to biweekly to support her bereavement process. Visit cadence was determined by evolving spiritual need within a longitudinal framework.<br>Interventions included theological reflection about her experience of faith, boundary clarification within family and church roles, grief processing (anticipatory and post-bereavement), and collaborative faith-integrated contemplative practice to stay resilient in her healthcare journey. The chaplain reinforced primary care and cardiology follow-ups and supported Mrs. A in emotionally processing her healthcare experiences post-visits.<br>Advance care planning discussions occurred after her mother's death, when Mrs. A began reflecting more directly on her own health trajectory and personal support system. These conversations were coordinated with her primary care clinician to ensure formal documentation in the medical record.
Referral to Supportive Resources	The chaplain facilitated a connection to a faith-based bereavement group aligned with Mrs. A's religious tradition.<br>In collaboration with the ambulatory social worker, screening for social determinants of health identified financial strain. This resulted in utility bill assistance and linkage to community-based financial resources.
Patient Experience and Outcomes	Over time, Mrs. A reported reduced spiritual distress, greater clarity and satisfaction in family and church roles, and improved follow-through with primary care and cardiology follow-ups. This case illustrates how TOC-integrated, digitally enabled spiritual care can address conflicted belief systems, grief, and isolation longitudinally within value-based ambulatory care.

Note.

*This case example is a composite representation of clinical experiences of patients in the spiritual health program and does not reflect the case or the medical record of any single, identifiable patient. Demographic and clinical details have been altered to ensure anonymity.
